# Synchronization of cytoplasmic and transferred mitochondrial ribosomal protein gene expression in land plants is linked to *Telo*-box motif enrichment

**DOI:** 10.1186/1471-2148-11-161

**Published:** 2011-06-13

**Authors:** Jie Wang, Yu Wang, Zhuo Wang, Lei Liu, Xin-Guang Zhu, Xiaotu Ma

**Affiliations:** 1 Key Laboratory of Systems Biology, Shanghai Institutes for Biological Sciences, Chinese Academy of Sciences, 320 Yueyang Road, Shanghai, China; 2 Key Laboratory of Computational Biology, CAS-MPG Partner Institute for Computational Biology, Shanghai Institutes of Biological Sciences, Chinese Academy of Sciences, Room 342, Physiology Building, 320 Yueyang Road, Shanghai, China; 3 School of Life Sciences and Biotechnology, Shanghai Jiao Tong University, 800 Dongchuan Road, Shanghai, China; 4 Institute of Plant Physiology and Ecology, Shanghai Institutes of Biological Sciences, Chinese Academy of Sciences, 300 Fenglin Road, Shanghai, China; 5 Shanghai Center for Systems Biomedicine, Shanghai Jiao Tong University, 800 Dongchuan Road, Shanghai, China; 6 Department of Molecular and Cell Biology, Center for Systems Biology, University of Texas at Dallas, 800 W. Campbell Road, Richardson, TX 75080, USA

## Abstract

**Background:**

Chloroplasts and mitochondria evolved from the endosymbionts of once free-living eubacteria, and they transferred most of their genes to the host nuclear genome during evolution. The mechanisms used by plants to coordinate the expression of such transferred genes, as well as other genes in the host nuclear genome, are still poorly understood.

**Results:**

In this paper, we use nuclear-encoded chloroplast (cpRPGs), as well as mitochondrial (mtRPGs) and cytoplasmic (euRPGs) ribosomal protein genes to study the coordination of gene expression between organelles and the host. Results show that the mtRPGs, but not the cpRPGs, exhibit strongly synchronized expression with euRPGs in all investigated land plants and that this phenomenon is linked to the presence of a *telo*-box DNA motif in the promoter regions of mtRPGs and euRPGs. This motif is also enriched in the promoter regions of genes involved in DNA replication. Sequence analysis further indicates that mtRPGs, in contrast to cpRPGs, acquired *telo*-box from the host nuclear genome.

**Conclusions:**

Based on our results, we propose a model of plant nuclear genome evolution where coordination of activities in mitochondria and chloroplast and other cellular functions, including cell cycle, might have served as a strong selection pressure for the differential acquisition of *telo*-box between mtRPGs and cpRPGs. This research also highlights the significance of physiological needs in shaping transcriptional regulatory evolution.

## Background

Mitochondria and chloroplasts evolved from the endosymbionts of once free-living α-proteobacteria and cyanobacteria, respectively [1]. Most of the genes in the endosymbionts were transferred to the nuclear genome during evolution, resulting in much smaller current organelle genomes than their ancient cousins [2-4]. Many transferred genes acquired promoters from their eukaryotic hosts [1], and most proteins expressed by these functional transferred genes were eventually translocated back to their organelle, guided by targeting peptides [4,5]. Intuitively, a tight coordination of gene expression between organelle genomes and the host nuclear genome should be important for cellular functions, as well as the overall fitness of the organism [6,7]. In fact, coordination between host and organelles through biochemical signaling has been extensively studied [8]. Genetically, the coordination between nuclear and mitochondrial genome expression [6], as well as the coordination between nuclear and chloroplast genome expression [7] has been investigated. However, the molecular mechanisms underlying the coordination between host and organelle functions are still far from understood. In this work, we aim to identify signals coordinating the expression of genes in mitochondrion, chloroplast and nucleus.

On the other hand, chloroplasts, mitochondria and the host cell all have protein translation machineries. In addition to the cytoplasmic ribosome of the host cell, mitochondria and chloroplasts each have their own respective ribosomes to translate proteins encoded in their genomes [9]. The existence of ribosomes in mitochondria and chloroplasts provides a certain freedom of independent biogenesis and/or development for these organelles [10,11]. Given that protein synthesis is required for organelle biogenesis, development and various biological processes in these different cellular compartments, investigation of the coordination of expression and regulation of ribosomal protein genes (RPGs) between host nucleus and each type of organelle can shed light on the coordination of organelle biogenesis, development and biological processes in these different compartments. Since most organelle RPGs were transferred to the nuclear genome during evolution (Figure S1 in Additional File 1), specialized *trans*-factors and *cis*-elements may have evolved to ensure the expression of these transferred RPGs in a coordinated manner. As such, identification of these factors or elements is critical to understanding the coordination of biogenesis and development between these different organelles. We thus chose to examine the expression and regulatory patterns of these transferred RPGs.

Our analysis show that the expression patterns of transferred mitochondrial (mtRPGs) and cytoplasmic (euRPGs) ribosomal protein genes are highly coordinated, while expressions of chloroplasts (cpRPGs) and euRPGs are not. This phenomenon appears in all investigated monocot and dicot plants, the expression datasets of which are available. By sequence analysis on the promoter regions of these RPGs, we identified a functional DNA motif, *telo*-box, which is linked to the observed differential coordination patterns. The *telo*-box is present in promoters of mtRPGs and euRPGs, but absent in promoters of cpRPGs across all examined land plants. The *telo*-box is also enriched in the promoter regions of genes encoding enzymes involved in DNA replication, indicating a potential role of *telo*-box in the cell cycle. Evidences from comparative genomics analysis indicated that the *telo*-box in mtRPGs was acquired from the host nuclear genome. Based on these results, we proposed a model of land plant nuclear genome evolution. In this model, after endosymbiosis, many genes in endosymbionts were transferred to the nuclear genome. The demand for a high-level coordination of energy supply might have been a strong selection pressure, which gradually led to coordinated expression of proteins in mitochondria with those involved in other cellular functions, including cell cycle.

## Results

In this section, we first examined the expression patterns of cytoplasmic, mitochondrial and chloroplast ribosomal protein genes (RPGs) using expression profiles across a wide array of tissues of *A. thaliana*, and confirmed our findings in several other monocot and dicot plants. Such comprehensive tissue-specific expression datasets provide an unbiased sampling of gene expression. Based on the striking co-expression between euRPGs and mtRPGs, but not between euRPGs and cpRPGs, we further studied the promoter sequences of these three sets of RPGs. We found a DNA motif known as *telo*-box in promoters of euRPGs and mtRPGs but not cpRPGs in all studied land plants, which explains the observed differential coordination patterns. Functional implication of *telo*-box and regulatory evolution of mtRPGs and cpRPGs after gene transfer is then studied.

### Co-expression between mtRPGs and euRPGs

**Figure 1:**
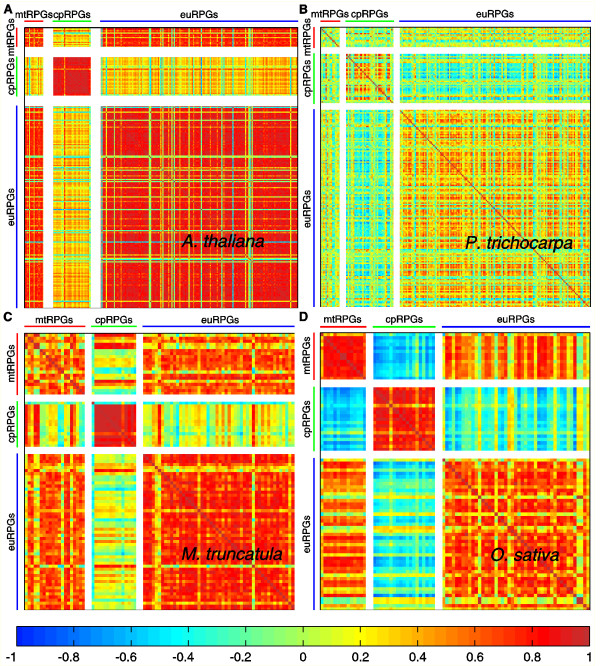
**Correlation between mRNA expression of mtRPGs, cpRPGs and euRPGs in four angiosperms**. The expression profiles of RPGs of *A. thaliana *(A), *P. trichocarpa *(B), *M. truncatula *(C) and *O. sativa *(D). Each element of the matrix represents the Pearson's correlation coefficient between the expression profiles of two RPGs. Color code is illustrated at the bottom panel.

Intuitively, biogenesis of cellular organelles needs to be highly coordinated to ensure the optimal growth of a plant cell and hence the organism. Since production of proteins is the key step for organelle biogenesis, the study of protein translational machinery, i.e., ribosomes, may provide insights on the coordination between host and the organelles. We therefore first asked how the expression of mtRPGs, euRPGs and cpRPGs are coordinated across various tissues in plants. Here we chose to use tissue-specific expression data to minimize possibility of spurious findings due to sample bias. Correlation analysis of the expression of cpRPGs, mtRPGs and euRPGs in *Arabidopsis thaliana *showed that the expression of mtRPGs was strongly positively correlated with the expression of euRPGs (Pearson's Correlation Coefficient, PCC = 0.6260 ± 0.3220, p < 1.0E-10, *t*-test). On the other hand, although the expression of cpRPGs showed significantly positive correlation with mtRPGs and euRPGs (PCC = 0.1582 4; 0.1901, p < 1.0E-10), the magnitude was much lower (p < 1.0E-10, Figure 1A). Similar results were also obtained in *Populus trichocarpa *(Figure 1B), *Medicago truncatula *(Figure 1C), and *Oryza sativa *(Figure 1D). Furthermore, the above observed correlation patterns were also found using tissue-specific protein expression level data in *A. thaliana *(Figure S2 in Additional File 1), where the protein expression levels between mtRPGs and euRPGs were significantly positively correlated (PCC = 0.1844 ± 0.4031, p = 4.8E-12), while the protein expression levels of cpRPGs were significantly negatively correlated with those of both mtRPGs and euRPGs (PCC = -0.1461 ± 0.4115, p = 1.4E-31). These results indicated that some regulatory mechanisms might exist for the differential coordination of expression between mtRPGs and cpRPGs.

The correlation of expression among RPGs was further examined in detail by using *A. thaliana *microarray data at several developmental stages (Figure S3 in Additional File 1). We found that the expression of mtRPGs and euRPGs was positively correlated across all developmental stages. In contrast, the correlation between the expression of cpRPGs with either mtRPGs or euRPGs changed dramatically during developmental progression. In particular, cpRPGs were found to be negatively correlated with mtRPGs and euRPGs at early developmental stages (7^th ^day: PCC = -0.2855 ± 0.3040, p < 1.0E-10 and 17^th ^day: PCC = -0.3166 ± 0.3086, p < 1.0E-10; Figure S3A, S3B in Additional File 1), but cpRPGs became positively correlated with mtRPGs and euRPGs at later developmental stages (21^st ^day: PCC = 0.3509 ± 0.2192, p < 1.0E-10; 8^th ^week: PCC = 0.3488 ± 0.5719, p < 1.0E-10; Figure S3C, S3D in Additional File 1). Notably, cpRPGs showed a much higher level of coordination in their expression levels at the 8^th ^week of seed development, as compared to the co-expression within either mtRPGs or euRPGs (within cpRPGs, PCC = 0.8681 ± 0.2332; within mtRPGs, PCC = 0.0361 ± 0.4613; within euRPGs, PCC = 0.3940 ± 0.3984; Figure S3D in Additional File 1).

### *Telo*-boxes are Enriched in Promoters of mtRPGs and euRPGs

**Figure 2:**
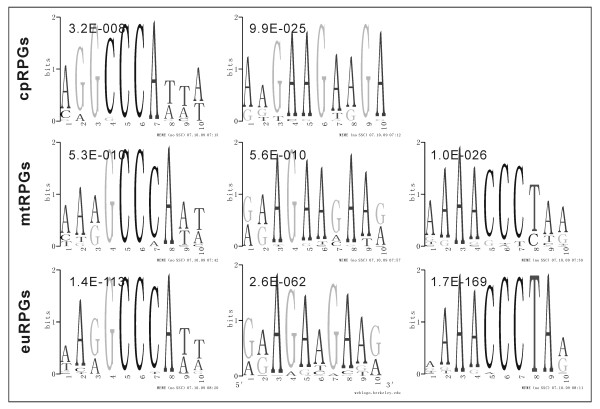
**Promoter motifs of cpRPGs, mtRPGs and euRPGs in *A. thaliana***. The first (GCCCA) and third (AAACCCT) motifs are known as site II motif and *telo*-box motif, respectively. The number on the upper left of each logo is the E-value of MEME prediction. Some motifs only enriched in certain classes of RPGs are shown in Figure S4 in Additional File 1.

**Figure 3:**
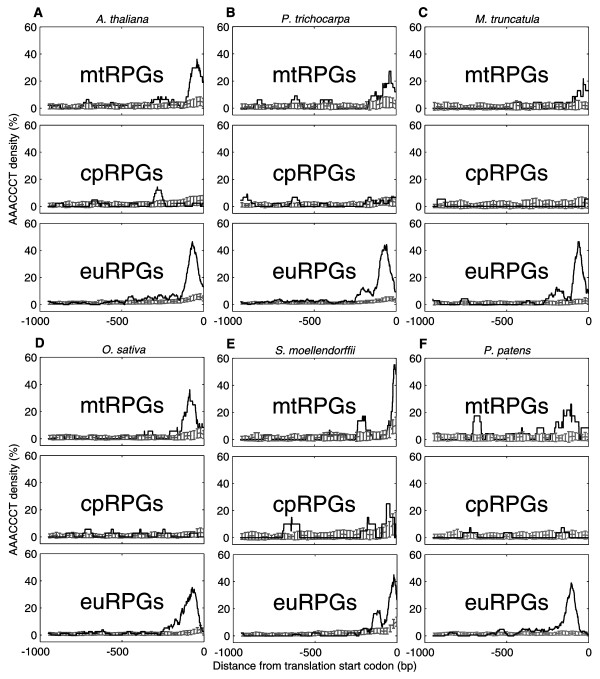
**Positional distribution of the *telo*-box motif (AAACCCT) of RPGs in all studied land plants**. *Telo*-box (dark solid line) is enriched in promoters of mtRPGs and euRPGs, but not in cpRPGs for *A. thaliana *(A), *P. trichocarpa *(B), *M. truncatula *(C), *O. sativa *(D), *S. moellendorffii *(E) and *P. patens *(F). Grey dashed line with error bar indicates motif density of background sequences. Window size is 50bp.

We next asked if the above observations could be explained by transcriptional regulatory elements. Analysis of the promoter sequences of nuclear-encoded cpRPGs, mtRPGs and euRPGs in *A. thaliana *using MEME [12] revealed conserved DNA motifs (Figure 2). The first motif, GCCCA, known as site II motif and highly enriched in promoters of all three classes of RPGs in *A. thaliana *(Figure 2), is a binding target of the transcription factor At-TCP20 [13]. The transcription factor corresponding to the second shared motif, GAAGAA, has not been identified. The third motif, AAACCCT, known as *telo*-box, is enriched in promoters of both euRPGs and mtRPGs, but not in promoters of cpRPGs in *A. thaliana *(Figure 2 and 3A). Similar results (Figure S5 in Additional File 1) were obtained using other DNA motif-finding tools e.g. AlignACE and DME [14,15]. Thus, the absence or presence of *telo*-box in RPG promoters was considered to be associated with the differential expression coordination patterns among cpRPGs, mtRPGs and euRPGs. In addition, mitochondrial and cytoplasmic RPGs with *telo*-box in their respective promoter regions showed significantly higher co-expression than those without *telo*-box (p < 0.001, Figure S6 in Additional File 1), further indicating the functional importance of *telo*-box in synchronizing the expression patterns of mtRPGs and euRPGs. In fact, such an association was also observed in three other angiosperm land plants, *P. trichocarpa*, *M. truncatula *and *O. sativa *(Figure 3B, 3C, 3D; though the appearance of *telo*-box in mtRPGs of Figure 3B and 3C is slightly less than that of Figure 3A and 3D, possibly due to noise in promoter annotation), for which both DNA sequence data and expression profiling data are available. In two recently sequenced land plant species, *Selaginella moellendorffii *and *Physcomitrella patens*, *telo*-boxes are also enriched in the promoter regions of corresponding mtRPGs and euRPGs, but not in cpRPGs (Figure 3E, 3F; the appearance of *telo*-box is slightly above background in cpRPGs of *S. moellendorffii*, possibly due to noise in promoter annotations). Furthermore, the chromosome location of *telo*-box is also consistently close to the translation start codon in all these examined plants (Figure 3), indicating that the positioning of *telo*-box may be functionally important for the proper expression of the regulated genes. The examined plant species covered a wide range of land plant species (including moss, spikemoss, monocot and dicot plants) (Figure S7 in Additional File 1); therefore, the association between the coordinated expression pattern of RPGs and the presence of *telo*-box pattern in the promoter regions of RPGs might be conserved in all land plants.

### *Telo*-boxes are Enriched in Promoters of Non-RPGs Highly Co-expressed with mtRPGs or euRPGs

**Figure 4:**
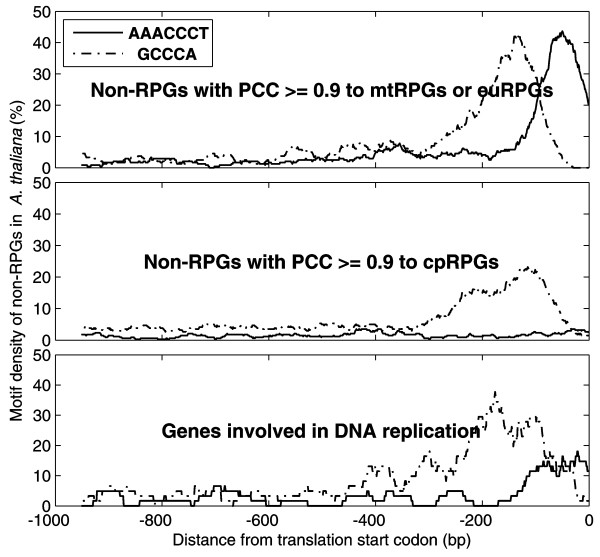
**Positional distribution of *telo*-box (AAACCCT) and Site II motif (GCCCA) of non-RPGs**. *Telo*-box (solid line) is enriched in genes involved in both DNA replication and non-RPGs (non-ribosomal protein genes) that are highly correlated with (PPC ≥ 0.9) mtRPGs or euRPGs, but not in non-RPGs that are highly correlated with (PPC ≥ 0.9) cpRPGs. Site II motif (dash-dot line) is enriched in all three groups.

We next examined whether *telo*-boxes are enriched in the promoter regions of non-ribosomal protein genes (non-RPGs) which are highly co-expressed with either mtRPGs or euRPGs in *A. thaliana*. In the identified 243 non-RPGs with PCC ≥ 0.9 to mtRPGs or euRPGs, the *telo*-box motif is significantly enriched in their promoter regions (p = 8.7E-63, Chi-square test, Figure 4). In contrast, in 341 non-RPGs which are highly co-expressed (PCC ≥ 0.9) with cpRPGs no significant enrichment of *telo*-box in their promoters is observed (Figure 4). This result further supports *telo*-box as the molecular mechanism underlying differential coordination patterns among cpRPGs, mtRPGs and euRPGs.

In the 243 non-RPGs highly co-expressed with mtRPGs or euRPGs (PCC ≥ 0.9), gene ontology analysis using DAVID [16] (on 127 genes with "biological process" annotation) indicates that most of these gene products are significantly related to "RNA processing and metabolism", "cell organization and biogenesis", and "protein translation and location" (Table 1). In addition, 140 genes with "molecular function" annotation are mostly related to "RNA, nucleotide, nucleic acid or protein binding", and "translation initiation factor activity" (Table 1).

**Table 1:** GO enrichment of non-RPGs highly correlated with mtRPGs or euRPGs

**NO**.*	GO term	**Count**^**§**^	**FDR**^**†**^
BP1	RNA processing	24	8.2E-9

BP 2	ncRNA metabolic process	19	2.3E-8

BP 3	protein folding	17	1.2E-6

BP 4	ribonucleoprotein complex biogenesis	16	8.9E-6

BP 5	mitochondrion organization	8	1.5E-5

BP 6	rRNA processing	12	1.8E-5

BP 7	ribosome biogenesis	15	4.9E-5

BP 8	ncRNA processing	13	1.6E-4

BP 9	chromatin organization	13	8.8E-4

BP 10	translational initiation	8	6.0E-3

BP 11	protein targeting to mitochondrion	5	2.4E-2

MF1	RNA binding	43	1.6E-9

MF2	nucleotide binding	68	2.5E-8

MF3	translation factor activity, nucleic acid binding	14	1.7E-6

MF4	translation initiation factor activity	12	3.5E-6

MF5	unfolded protein binding	12	7.8E-6

* BP: Biological process, MF: Molecular function. ^§ ^The total numbers of annotated genes in GO biological process and molecular function are 127 and 140, respectively. ^† ^P-value is corrected by False Discovery Rate (FDR) using DAVID [16].

Since it has been shown in *Drosophila *[17] that euRPGs are regulated by a transcription factor, DREF, which participates in DNA replication, and since the expression of ribosomal protein genes is related to cell proliferation [18], we next studied whether DNA replication genes (e.g., origin recognition, replicative helicases, helicase loading factors) in *A. thaliana *had coordinated expression with mtRPGs/euRPGs and showed enriched *telo*-boxes in their promoters. Interestingly, our analysis revealed that expression of these DNA replication genes was also highly positively correlated with that of mtRPGs/euRPGs (PCC = 0.5643 ± 0.2998). In addition, these genes have significantly enriched *telo*-boxes in their promoter regions (p = 3.8E-6, Chi-square test, Figure 4). Therefore, the shared regulation between ribosomal protein genes and DNA replication genes are conserved between insects and plants.

### Conservation of Transcription Factor Purα that Binds to *Telo*-box

Given the coordinated expression pattern between mtRPGs and euRPGs, and the common *telo*-box in promoters of mtRPGs and euRPGs, it will be interesting to ask whether the *trans*-factor of *telo*-box is conserved among the studied species. We therefore studied the conservation pattern of transcription factor Purα, which is known to recognize *telo*-box [19-21]. First, the homolog of Purα was found to be present in all the examined land plants. Secondly, multiple sequence alignment for the Purα protein (Figure S8 in Additional File 1) reveals that several domains are highly conserved in all studied land plants, including the DNA-binding domain [21]. This observation is consistent with the highly conserved sequence of *telo*-boxes in land plants (Figure 3), and provides further support that *telo*-box might be a controlling mechanism of the coordinated expression between mtRPGs and euRPGs. In addition, this result indicates that Purα may participate in regulating the biogenesis and development of mitochondria.

### Evolutionary Origin of *Telo*-box in Promoters of Transferred mtRPGs

Mitochondrial RPGs may have acquired *telo*-boxes for their coordinated expression with euRPGs in one of two ways: (1) they acquired *telo*-boxes after transferring into the nuclear genome or (2) they possessed *telo*-boxes in the endosymbionts, and the regulatory regions were carried on during transfer. To study these hypotheses, we searched genomic sequences of mitochondrion and chloroplast ancestors, respectively. Our result indicated that neither proto-mitochondrial ancestor (*Rickettsia prowazekii str. Madrid E *[22,23]) nor proto-chloroplast ancestor (*Synechocystis sp. PCC6803 *[4,24]) contained *telo*-boxes (data not shown). In addition, to account for the possibility that *telo*-boxes may have been lost during the evolution of *R. prowazekii *and *Synechocystis*, we also searched all available 132 chloroplast genomes and 25 plant mitochondria genomes, and *telo*-box was still not found (data not shown). These results indicated that mtRPGs acquired *telo*-box after endosymbiosis, whereas cpRPGs did not either successfully acquire or keep the *telo*-box after endosymbiosis.

Given the above results, it is interesting to ask if these transferred mtRPGs happened to be inserted into nuclear genomic regions where *telo*-box was enriched. To test this hypothesis, we first studied the appearances of *telo*-boxes in the vicinity of mtRPGs and cpRPGs (between upstream 40kb and downstream 40kb of the translation start codon). As can be seen in Figure S9A in Additional File 1, no significant difference of *telo*-box enrichment in flanking sequences between mtRPGs and cpRPGs was found. This result indicates that biased insertion during gene transfer between mtRPGs and cpRPGs is unlikely. To further confirm this conclusion, we studied the distances from each mtRPG/cpRPG to the closest non-ribosomal nuclear gene in the same chromosome which has *telo*-box in its promoter region, as these non-ribosomal nuclear genes might provide source of *telo*-boxes for transferred mtRPGs. As seen in Figure S9B in Additional File 1, no significant difference was observed between mtRPGs and cpRPGs in their distances to their respective closest non-RPG neighbors regulated by *telo*-boxes (p > 0.1, *t*-test for the mean distances of mtRPGs and cpRPGs to the closest non-RPG neighbors with *telo*-box). Taken together, these results indicate that selective pressures, rather than preferential insertion regions, may be the reason for mtRPGs and cpRPGs to acquire different regulatory mechanisms to coordinate their biogenesis with host after gene transfer.

### The Coordination between mtRPGs and euRPGs is Land Plant-Specific

To check whether the coordinated pattern between mtRPGs and euRPGs is unique in land plants, we studied whether this pattern also existed in algae. We chose to study the brown algae *Ectocarpus siliculosus*, which is phylogenetically distant from land plants [25] (Figure S7 in Additional File 1) and genome sequence and gene expression profiling data of which are available [25,26]. Analysis on RPGs of *E. siliculosus *indicated that *telo*-boxes were not enriched in promoters of any types of RPGs (data not shown) and that the expression of mtRPGs is clearly independent from that of euRPGs (PCC = -0.0365 ± 0.2127, p > 0.01; Figure S10A in Additional File 1). This result indicated that the coordination of mtRPGs and euRPGs might be land plant-specific. To further confirm this conclusion, we used the unicellular green algae *Chlamydomonas reinhardtii *which diverged from land plants over a billion years ago [27]. We separately measured the expression levels of 6, 7 and 10 highly reliable mtRPGs, cpRPGs and euRPGs of *C. reinhardtii *under four conditions, including continuous light, continuous dark, high and low nitrogen treatments (see Methods). Results indicate that the expression levels of mtRPGs and euRPGs are not coordinated (Figure S10B in Additional File 1); furthermore, RPGs in *C. reinhardtii *lack *telo*-box motifs (data not shown). Taken together, these results indicate that the differential transcriptional modulation of cpRPGs and mtRPGs by *telo*-box is land plant-specific.

### Regulatory Changes are Common for RPGs in other Species

In addition to the mitochondrion shared by all eukaryotes, plants, as compared to the animal species, have chloroplasts. Therefore, the transcriptional evolution of plant organelle ribosomal proteins may have been more complicated than other species. As demonstrated in this work, the acquisition of *telo*-boxes of transferred genes after endosymbiosis is different between mtRPGs and cpRPGs. In fact, such a dramatic change in the transcriptional regulation of ribosomal protein genes has already been seen in other organisms. For example, the *cis*-elements for cytoplasmic ribosomal protein genes are found to be significantly different among fungi, insects and mammals [17]. It was also reported that the ribosomal regulation is highly evolvable in yeast through the use of an intermediate redundant regulatory program [28]. Most strikingly, it was shown that the loss of a *cis*-element AATTTT in promoters of mtRPGs following whole-genome duplication is linked to rapid anaerobic growth of *S. cerevisiae *[29]. However, unlike the above-mentioned discoveries, our results highlight the differential acquisition of *cis*-elements after gene transfer, possibly due to the different physiological needs between mitochondria and chloroplast. Taken together, these discoveries indicate that the gene expression regulatory programs are highly evolvable for ribosomal protein genes, which are one of the most conserved gene families among all kingdoms. Therefore it will not be surprise to see dramatic changes in regulatory programs in other less conserved gene families which are specific to certain species. In fact, our discovery adds more support for the view that speciation primarily arise from changes in gene regulatory regions [30].

## Discussion

### The co-expression pattern of RPGs in different cellular compartments and the regulatory elements controlling this pattern

Analysis of the expression pattern of genes involved in ribosomal proteins in different compartments showed that mtRPGs exhibited a high level of co-expression with euRPGs, while cpRPGs did not show such a high level of co-expression with euRPGs (Figure 1). This pattern of expression was conserved across different land plant species examined in this study (Figure 1, Figure S2 in Additional File 1). These results indicated that the strong coordination of expression of mtRPGs and euRPGs possibly increased plant fitness, although the detailed mechanisms have not yet been elucidated. Interestingly, although the expression of cpRPGs was not well correlated with euRPGs when data from different developmental stages were pooled together in the analysis, cpRPG expression showed negative correlation with expression of mtRPGs and euRPGs in the early developmental stage in *A. thaliana *(Figure S3A, S3B in Additional File 1). In contrast, at the later developmental stages of *A. thaliana*, cpRPGs showed a positive correlation with expression of euRPGs and mtRPGs (Figure S3C, S3D in Additional File 1). Thus, while the expression levels of mtRPGs showed a strongly positive correlation with euRPGs under different developmental stages, the relationship between the expression of cpRPGs and euRPGs was developmental stage-dependent. Since expression data at developmental stage resolution are only available for *Arabidopsis*, it will be interesting to see if similar observations can be made in more plant species. Nonetheless, since the plants species we studied give a very good representation of the land plants (Figure S7 in Additional File 1), it is highly likely that the differential modulation of the transcriptional regulation of mtRPGs, cpRPGs, and euRPGs is a ubiquitous phenomenon for all land plants. However, the physiological significance of these patterns awaits further investigation.

Analyses of the promoter regions of RPGs help identify the *cis*-regulatory motifs potentially responsible for the different patterns of co-expression between RPGs in different cellular compartments (Figure 1). Three distinct *cis*-regulatory motifs were identified, two shared by mtRPGs, cpRPGs and euRPGs and one only existing in mtRPGs and euRPGs (Figure 2). The first motif, GCCCA, is called a site II motif, which is the binding site of a transcription factor known as At-TCP20 [31]. At-TCP20 is expressed in many different tissues in *A. thaliana *and can influence cell division and growth coordination [31]. The third motif, AAACCCT, called *telo*-box, is the binding site of the Purα transcription factor, which has been suggested to be a partner of the At-TCP20 [20]. Our analysis provided additional evidence for this viewpoint, since the *telo*-box is in close proximity to the site II motif in promoter regions of the RPGs (Figure S11 in Additional File 1). Therefore, both *cis*-elements could work as a module to coordinate gene expression, or they could also participate in controlling the cell cycle. The function of the second motif, GAAGAA, is not clear at this point, but it is preferentially enriched in chromosomal locations close to the other motifs (Figure S11 in Additional File 1), which indicates that its function might also be related to cell cycle control. Interestingly, the identified promoter motifs do not seem to be shared with either mammals or insects [17,32]. This result indicates that the regulatory mechanisms of ribosomal protein genes, one of the most conserved gene families, are highly evolvable and highlights the contribution of regulatory network changes in evolution, in addition to the contribution of gene sequences.

### The special role of *telo*-box in coordinating DNA, protein synthesis, energy production and cell cycle

*Telo*-box, which is the binding site of the Purα transcription factor, clearly does not exist in chloroplasts (Figure 2 and 3). This motif (AAACCCT or AACCCTA) is homologous to a telomere repeat (AAACCCT)_n _of land plants, which is enriched in the ends of chromosomes [33]. *Telo*-box was first observed in promoters of translation elongation factor eEF1A [34-37] and subsequently found within the promoters of PCNA (proliferating cell nuclear antigen) and RNR (ribonucleotide reductase), both of which are over-expressed in cycling cells [19]. Our analysis further showed that *telo*-box was enriched in genes involved in nucleotide (DNA or RNA), protein binding and in processes ranging from "cell organization and biogenesis", "RNA processing and metabolism", to "protein translation and location" (Table 1). These results indicated that the *telo*-box motif likely functioned at the top of the hierarchy coordinating host and mitochondrion in these different processes.

Furthermore, this study indicated that the *telo*-box might be a major regulator of cell cycle activity, which is supported by the following evidences: 1) The *telo-box *motif is enriched in mtRPGs, euRPGs and genes of DNA replication machinery; 2) DNA replication, protein synthesis, and energy production by mitochondria are all required for normal cell cycle [38]; 3) cells need to synthesize large amounts of DNA and protein in order to increase cell size before mitosis [39,40]; 4) the transcription factor, At-Purα, which is a *trans*-factor for the *telo*-box, controls both gene transcription and DNA replication [41].

### A hypothetical model for the differential acquisition of *telo*-box during organelle evolution

Both mitochondria and chloroplasts are the descendents of endosymbionts; however, mtRPGs showed a high level of co-expression with euRPGs, while cpRPGs did not (Figure 1). We have linked this phenomenon to a lack of *telo*-box in the promoter regions of cpRPGs (Figure 3), which indicates the possibility that *telo*-box might be the critical binding motif contributing to the difference in the expression of RPGs in different cellular compartments. This, in turn, has raised a number of important questions.

First, what is the origin of *telo*-box? The fact that the genomic sequence of ancestors of mitochondrion and chloroplast did not have *telo*-box indicated that mtRPGs acquired and successfully maintained the *telo*-box after endosymbiosis, while cpRPGs either did not acquire or failed to maintain the *telo*-box during the evolutionary process after endosymbiosis. Although cpRPGs have a relatively short evolutionary span (1.2~1.5 Ga) compared to mtRPGs (>1.5 Ga) [1], it is unlikely that the cpRPGs never acquired *telo*-box. In fact, after endosymbiosis, most of the genes in the endosymbionts' genome were transferred to the host nuclear genome [4,42,43] and formed a unique metabolic network of the current chloroplast [44,45]. Most of these genes have acquired new *cis*-regulatory motifs in their promoter sequences (Figure S12 in Additional File 1).

Secondly, why did cpRPGs fail to maintain the *telo*-box during evolution? Although *telo*-box could have been integrated into the promoter regions of cpRPGs, these regulatory elements were clearly selectively purged out after gene transfer. This indicates that a strong negative selection pressure may have resulted from simultaneous expression of cpRPGs with those of mtRPGs and euRPGs. One possible mechanism of this negative selection pressure is that photosynthesis generates oxygen, which can potentially generate reactive oxygen species under high light [46]. Reactive oxygen species, such as superoxide, not only cause direct damage to DNA [47], but also influence structure and, correspondingly, the function of proteins [48,49], including proteins involved in DNA replication and protein synthesis. As a result, it is disadvantageous to have photosynthesis occur simultaneously with the DNA replication and protein synthesis, which are required for normal cell cycle. Indeed, DNA replication usually occurs at midnight, quite possibly to avoid damaging DNA by UV radiation [50,51]. Therefore, the potential damage caused by concurrence of photosynthesis and processes related to cell cycle might have generated a strong negative selection pressure, which purged the *telo*-box from the cpRPGs.

Thirdly, what is the reason for the strong coordinated expression of mtRPGs and euRPGs? In all the examined land plant species, the expressions of mtRPGs and euRPGs are highly co-expressed (Figure 1). This co-expression does not seem to be dependent on developmental stage or growth conditions (Figure S3 in Additional File 1). This fact indicates that there is a strong positive selection for co-expression of mtRPGs and euRPGs. Again, the clue may come from the cell cycle. Two of the three conserved binding sites, Site II and *telo*-box, are related to cell cycle control [13,20,31]. Furthermore, the genes involved in DNA replication are highly positively correlated with mtRPGs and euRPGs and also harbor enriched Site II and *telo*-box motif in their promoters (Figure 4). In addition, the genes highly co-expressed with euRPGs or mtRPGs are enriched in nucleotide (DNA or RNA) and protein binding function (Table 1). This indicates that cell cycle might be coordinated with mitochondrial function. To enable a cell go through cell cycle, cells need to have large amounts of protein synthesized and have DNA replicated in order to reach a certain cell size [40]. Protein synthesis and DNA replication requires energy, which will be supplied by mitochondria. Therefore, a highly coordinated function of mitochondria and cell cycle might have created a positive selection pressure, which facilitated the maintenance of *telo*-box after mtRPGs gained it from the host nuclear genome.

**Figure 5:**
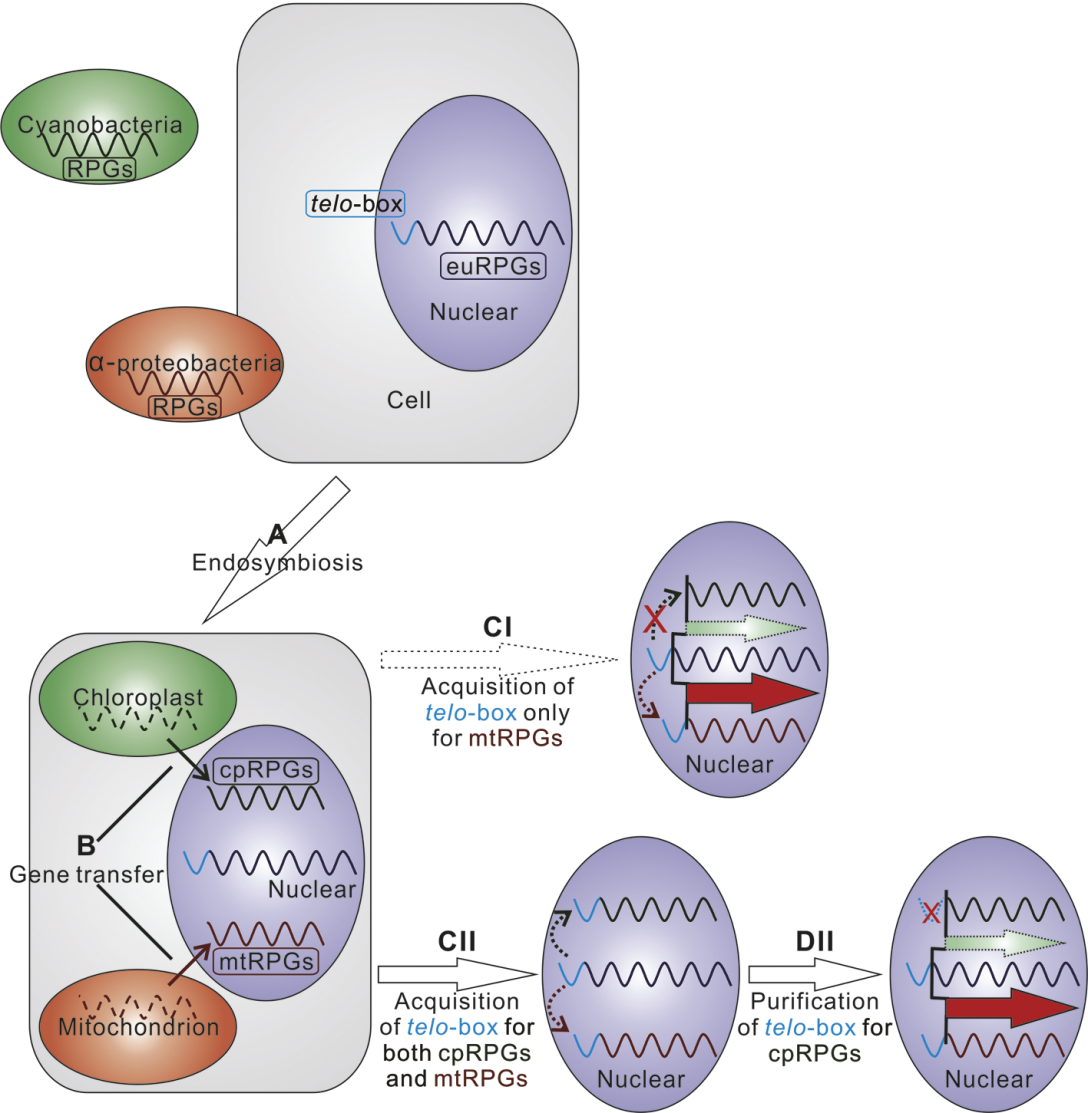
**The model of plant nuclear genome evolution**. (A) α-proteobacteria and cyanobacteria with RPGs not containing *telo*-box motif (purple curve) were engulfed in host cell, which possesses euRPGs with *telo*-boxes. (B) RPGs of endosymbionts (chloroplast and mitochondrion) are transferred into host nuclear genome. (CI) mtRPGs, but not cpRPGs, acquired *telo*-boxes from host nuclear genome. (CII) both mtRPGs and cpRPGs acquired *telo*-boxes from host nuclear genome. (DII) under negative selection pressures, *telo*-boxes of cpRPGs are purged. As a result, mtRPGs and euRPGs share *telo*-box and exhibit synchronized expression (thick red arrow). On the other hand, cpRPGs do not have *telo*-boxes in their promoter regions, and the expression coordination between cpRPGs and euRPGs is weak (dashed green arrow).

Following the above reasoning, we proposed a model to explain the evolution of the promoter structure in mitochondria and chloroplast (Figure 5). In this model, after endosymbiosis, endosymbionts transferred most of their genes into the host nuclear genome. The transferred genes, in turn, acquired regulatory elements, including the *telo*-box, from the nuclear genome. During the evolutionary process, DNA damage by the reactive oxygen species from aberrant cpRPG expression created a negative selection pressure and purged *telo*-box from the cpRPGs, while, on the other hand, the high level of coordination between mitochondria function and cell cycle created a positive selection force, thus maintaining the *telo*-box in mtRPGs. Therefore, the selective removal or maintenance of *telo*-box in RPGs by possibly different mechanisms, one being negative selection force and another being positive selection force, created the dramatic differences we found in the expression pattern of these two organelles. Furthermore, these negative and positive selection forces might have been major forces in shaping the evolution of promoter structure in these two important organelles in plant cell.

## Conclusions

This study showed that mtRPGs, but not cpRPGs, displayed strongly correlated expression with euRPGs in land plants. This phenomenon is linked to a highly conserved *cis*-regulatory element, AAACCCT, known as the *telo*-box motif, which is present in promoters of cytoplasmic and mitochondrial RPGs, but not in cpRPGs. Considering the fact that the *telo*-box is also enriched in promoters of genes involved in DNA replication, it seems likely that coordination of mitochondria function (mainly ATP production) with other cellular functions might have been a strong positive selection pressure in shaping the genome structure of land plants. Similarly, the potential damage caused by the concurrence of photosynthesis and cell cycle might have created a strong negative selection pressure which purged *telo*-box from the promoters of cpRPGs. This study indicated that the gain and loss of a single *cis-*element, possibly by different reasons, could result in dramatic differences in transcriptional regulation between chloroplast and mitochondria in land plants (Figure 5).

## Methods

### Species

Five plant species with both gene expression data and genome sequence data available are included in this study: *Arabidopsis thaliana *(mouse-ear cress), *Populus trichocarpa *(black cottonwood), *Medicago truncatula *(barrel medic), *Oryza sativa *(rice) and *Ectocarpus siliculosus *(brown algae). Two other recently sequenced species, *Selaginella moellendorffii *(spikemoss) and *Physcomitrella patens *(moss), are also included in our analysis to demonstrate the conservation of discovered DNA motifs. In addition, we also include the green algae *Chlamydomonas reinhardtii*, which has complete genome sequence and gene expression data, although, unfortunately, its micro-array did not include mtRPGs. We thus measured the expression levels of its ribosomal protein genes by RT-PCR experiments as described in the section subtitled "RT-PCR experiment for RPGs in *C. reinhardtii*". The evolutionary relationship of the studied species is shown in Figure S7 in Additional File 1[25,52,53].

### Catalogs of cytoplasmic and transferred organelle ribosomal protein genes

Sequence information for *A. thaliana*, *M. truncatula *and *E. siliculosus *was downloaded from http://www.ncbi.nlm.nih.gov/http://www.medicago.org/index.php and http://bioinformatics.psb.ugent.be/webtools/bogas/[25], respectively. Sequences for *P. trichocarpa*, *O. sativa*, *S. moellendorffii*, *P. patens *and *C. reinhardtii *were obtained from the U.S. Department of Energy Joint Genome Institute (http://genome.jgi-psf.org/). To collect a full catalog of cytoplasmic and transferred organelle ribosomal protein genes, BLASTP was used to search the protein sequences of all studied species, using E-value of 10^-5 ^as the significance cutoff. To obtain nuclear-encoded cpRPGs and mtRPGs, ribosomal proteins encoded in Syn (*Synechocystis sp. PCC6803*, current-day cyanobacteria as proto-chloroplast ancestor [22,23]) and Rpr (*Rickettsia prowazekii str. Madrid E*, current-day α-proteobacteria as proto-mitochondrial ancestor [4,24]) were used as query sequences, respectively. However, some genes in Syn and Rpr may have been lost since the endosymbiosis events. Therefore, we further collected ribosomal protein genes still present in any of the currently available plant chloroplast (132 plant species) and mitochondrial (25 plant species) genomes to account for the above concern. In *A. thaliana*, cytoplasmic ribosomal protein genes (euRPGs) were obtained from NCBI. These well annotated euRPGs were further used to annotate euRPGs in other species (provided as Additional File 2).

We then used TargetP [54] to predict the cellular localization of the above collected proteins in each species. Since proteins were predicted to be targeted to a specific organelle with reliability class ≥ 3 are considered to be highly reliable [54], here only proteins with E-value < 10^-5 ^by BLASTP and reliability class ≥ 3 by TargetP were selected as RPGs for corresponding organelles, respectively. For *C*. *reinhardtii*, euRPGs were obtained from the Ribosomal Protein Gene database [55], and cpRPGs were identified using the experimental results [56,57]. The promoter sequences and expression datasets of ribosomal protein genes for each class in each species are summarized in Additional File 2 and Additional File 3, respectively. The catalog of cytoplasmic and transferred organelle ribosomal protein genes is also provided in Table S1 in Additional File 1.

### Gene expression data

To study the expression characteristics of ribosomal protein genes, large-scale microarray expression datasets were collected. The gene expression profile focusing on *A. thaliana *development [58], which includes various tissue samples across 4 developmental stages, i.e., 7^th^, 17^th^, 21^st ^day and 8^th ^week, was downloaded from http://www.weigelworld.org. The gene expression data for *O. sativa *and *M. truncatula *were downloaded from http://www.plexdb.org/. The gene expression data with platform number GPL5921 and GPL963 were downloaded from NCBI GEO for *P. trichocarpa *and the green algae *C. reinhardtii*, respectively. The gene expression data for *E. siliculosus *were downloaded from http://www.ebi.ac.uk/microarray-as/ae/ (accession numbers: E-TABM-578 [26]). Each array was first standardized to have a mean value of 0 and a standard deviation of 1. Probe sets corresponding to the same gene were collapsed to a single number by taking the mean value. Expression data for ribosomal protein genes were then extracted for further expression analysis (provided as Additional File 3). The gene expression data for special developmental stages in *A. thaliana *were extracted from whole expression data according to developmental stages of samples, as described [58]. In addition, tissue-specific protein expression level data for *A. thaliana *were obtained from http://fgcz-atproteome.unizh.ch/[59]. We used Pearson's Correlation Coefficient (PCC) to measure the co-expression between gene/protein pairs.

### Promoter sequences of RPGs

To analyze the transcriptional regulatory mechanism of cpRPGs, mtRPGs and euRPGs, we retrieved the promoter sequences (upstream 1kb relative to the translation start codon) for each ribosomal protein gene in each species (also provided as Additional File 2). These sequences were further used to search for potential *cis*-regulatory motifs using the MEME software with parameters of 5-10 in width for motif discovery [12]. To avoid possible bias in motif discovery, other tools including AlignACE [14] and DME [15] were also used. Motif width was set to be 10 for both software, and the background sequences for DME were the promoters of non-RPGs. Background sequences used in Figure 3 were randomly selected from promoters (upstream 1kb) of genes except RPGs in each species. We bootstrapped fifty datasets, each consisting of the same number as the set of cpRPGs, mtRPGs and euRPGs, and then took the mean value and standard deviation as the motif density and corresponding errors of background sequences, respectively. The distances between RPGs and non-RPGs with *telo*-box were calculated according to their translation start codon. The non-RPGs located in the same chromosome and transcribed at the same orientation with RPGs were choose for calculating the shortest distances.

### RT-PCR experiment for RPGs in *C. reinhardtii*

*C. reinhardtii *strain CC-503 cw92 mt+ [60] was cultured in liquid TAP medium [61] at 25°C under continuous light or dark. NH_4_Cl concentration in TAP medium (7 mM) was increased five-fold for the high nitrogen treatment and reduced ten-fold for the low nitrogen treatment. There were at least three replicates for each condition. Cells were collected at their mid-exponential phase of growth by centrifugation (4000 × g for 3 min). Isolation of total RNA was performed with the Triazol reagent (Invitrogen) according to instructions of the manufacturer. After DNase treatment, single-stranded cDNA was synthesized from total RNA according to the manual of PrimeScript II 1st Strand cDNA Synthesis Kit (TaKaRa) and used as templates for real-time PCR reactions. Real-time PCR was performed on the LightCycler^® ^instrument (Bio-Rad CFX96 Real-time PCR Detection System) using SYBR Green as a fluorescent dye (iQ SYBR Green Supermix, Bio-Rad; 2x mixture contains 100 mM KCl, 40 mM Tris-HCl, pH 8.4, 0.4 mM each dNTP, 50 U/ml iTaq DNA polymerase, 6 mM MgCl_2_, SYBR Green I and 20 Nm fluorescein). Each individual reaction contains 1.0 pmole of the indicated primers (provided in Additional File 4) and 1 μl of 5-fold diluted single-stranded cDNA. The final volume of each reaction was 20 μl. PCR conditions were as follows: 10 min at 95°C for activation of the hot start Taq polymerase and 40 cycles for the melting (30s at 95°C), annealing (30s at 60°C) and extension (30s at 72°C). The fluorescence measurement was made at the end of the annealing step. The Ubiquitin ligase (*Chlamydomonas *GenBank ESTs: BU648530, BE237749, BE237718, BU648531) was used as the housekeeping gene ([62], the primer sequences are provided as Additional File 4). Expression of this gene was previously shown to be constitutive under the different conditions used [63]. For each condition and gene, we first filtered the undetected values, calculated the mean value of CT (cycle threshold), and then normalize the expression value with formula: 2^[Mean value (CT) - Control value]/Control value^. The resulted value was used as expression level for the analysis of expression correlation by calculating Pearson's Correlation Coefficient (PCC) of each gene pair (provided as Additional File 3).

## List of Abbreviations

RPGs: Ribosomal Protein Genes; mtRPGs: mitochondrial RPGs; cpRPGs: chloroplast RPGs; euRPGs: cytoplasmic RPGs; non-RPGs: non-Ribosomal Protein Genes; PCC: Pearson's Correlation Coefficient.

## Authors' contributions

JW and XM collected data. YW and XZ performed experiments on *C. reinhardtii*. JW, YW, ZW, LL, XZ and XM analyzed the data and wrote the manuscript. XZ proposed the evolutionary model to explain the data. XM conceived of the study. All authors read and approved the final manuscript.

## Supplementary Material

Additional file 1 **Table S1 and Figures S1-S12**. **Table S1**: Catalogs of cytoplasmic and transferred organelle ribosomal protein genes. **Figure S1**: Occurrence frequency of RPGs in 132 plant chloroplast and 25 plant mitochondrial genomes. (A) More than half of chloroplast RPGs are absent in most chloroplast genomes of 132 plants surveyed (green dash line), providing a raw estimate of the lower bound of transfer frequency of cpRPGs. (B) Similarly, there are over 50 RPGs in *Rickettsia prowazekii str. Madrid E *(α-proteobacteria, an ancient cousin of mitochondrion), whereas only 18 mtRPGs can be found in the union of all 25 plant mitochondrial genomes and most mtRPGs are absent in over half studied species (red dash line). Genes with zero occurrence frequency are present in corresponding ancient cousin but absent in chloroplast/mitochondria of all studied plants. **Figure S2**: Protein expression correlation of mtRPGs, cpRPGs and euRPGs in *A. thaliana*. Each element of the matrix represents the Pearson's correlation coefficient between the expression profiles of two ribosomal proteins. **Figure S3**: RNA expression correlation of mtRPGs, cpRPGs and euRPGs in different tissues of several developmental stages in A. thaliana. 7^th ^day (A), 17^th ^day (B), 21^st ^day (C) and 8^th ^week (D) corresponds to stage of seedling, leaf, flower and seed of *A. thaliana*, respectively. Each element of the matrix represents the Pearson's correlation coefficient of the expression profiles of each two RPGs. Color code is illustrated in bottom panel. **Figure S4**: Putative promoter motifs only enriched in one of cpRPGs, mtRPGs, and euRPGs in *A. thaliana*. The number on the left of each logo is E-value of MEME prediction. **Figure S5**: Putative promoter motifs predicted by AlignACE and DME. The number on the left of each logo is score of AlignACE and DME prediction. **Figure S6**: Expression correlation between RPGs with *telo*-box and those without *telo*-box. *Telo*-box here indicates the sequence AAACCCT or AACCCTA. The error-bar is the standard deviation of expression correlation. A symbol of the three stars (***) indicates the p-value is less than 0.001. **Figure S7**: The phylogenetic relationship of all investigated species. The phylogenetic relationship of angiospermae is derived according to the 16S rRNA of chloroplast genome in each species, using the neighbor-joining method. Both gene expression data and genome sequence data are available for *O. sativa*, *A. thaliana*, *P. trichocarpa*, *M. truncatula *and *E. siliculosus*, whereas only genome sequence data are available for *P. patens *and *S. moellendorffii*. The current gene expression data for *C. reinhardtii *do not include mtRPGs. Gray fonts indicate species that are not sequenced yet. Branch lengths are not scaled to time. **Figure S8**: Alignment of Purα proteins in all studied land plants. Some species have two Purα proteins. Here three highly conserved domains of Purα are shown. The tree on the left is drawn based on the sequences of Purα proteins with neighbor-joining method. The sequence labeled with the cyan line is involved in DNA-binding. Asterisks, colons, and dots indicate identical (red), strongly similar (green) and weakly similar (blue) residues, respectively. **Figure S9: The occurrence of *telo*-boxes (A) and non-RPGs with *telo*-box (B) relative to mtRPGs or cpRPGs**: (A) The star symbol indicates the significance of the occurrence within -1kb for mtRPGs. (B) The insets indicates mean distances of mtRPGs (cyan) and cpRPGs (magenta) relative to their closest upstream (left) or downstream (right) non-ribosomal protein genes (non-RPGs) with *telo*-box, respectively. The error-bar in the insets is the standard deviation of the distances. **Figure S10**: RNA Expression correlation of mtRPGs, cpRPGs and euRPGs in brown (A) and green (B) algae. Each element of the matrix represents the Pearson's correlation coefficient between the expression profiles of two RPGs. RNA expression level is measured by RT-PCR experiment for green algae *C. reinhardtii *(see Methods). **Figure S11**: Positional distribution of Site II, GAAGAA and *telo*-box of RPGs in *A. thaliana*. Site II motif (GCCCA, magenta line), GAAGAA (dark green line) and *telo*-box motif (AAACCCT, cyan line) are close to each other in promoter regions of RPGs. **Figure S12**: Promoter motifs of nuclear-encoded chloroplast genes in *A. thaliana*. (A) The logos of the motifs are predicted with MEME. The upper-left number of each logo is E-value. (B) The motif density of nuclear-encoded chloroplast genes (NCGs) in *A. thaliana *(Ath) and corresponding genes in *Synechocystis sp. PCC6803 *(Syn). The nuclear-encoded chloroplast genes in *A. thaliana *are identified by Martin et al. (2002). Click here for file

Additional file 2 The promoter sequences of RPGs for studied species Click here for file

Additional file 3 The expression levels of RPGs for studied species Click here for file

Additional file 4 The primers of RPGs for RT-PCR experiment in *C. reinhardtii* Click here for file

## References

[B1] Dyall SD Brown MT Johnson PJ Ancient invasions: from endosymbionts to organelles Science 2004 304 253 257 10.1126/science.1094884 15073369

[B2] Glockner G Rosenthal A Valentin K The structure and gene repertoire of an ancient red algal plastid genome J Mol Evol 2000 51 382 390 11040290 10.1007/s002390010101

[B3] Gray MW Burger G Lang BF Mitochondrial evolution Science 1999 283 1476 1481 10.1126/science.283.5407.1476 10066161

[B4] Martin W Rujan T Richly E Hansen A Cornelsen S Lins T Leister D Stoebe B Hasegawa M Penny D Evolutionary analysis of *Arabidopsis*, cyanobacterial, and chloroplast genomes reveals plastid phylogeny and thousands of cyanobacterial genes in the nucleus Proc Natl Acad Sci USA 2002 99 12246 12251 10.1073/pnas.182432999 12218172 PMC129430

[B5] Millar AH Heazlewood JL Kristensen B Bruan HP Moller IM The Plant Mitochondrial Proteome Trends Plant Sci 2005 10 36 43 10.1016/j.tplants.2004.12.002 15642522

[B6] Giege P Sweetlove LJ Cognat V Leaver CJ Coordination of Nuclear and Mitochondrial Genome Expression during Mitochondrial Biogenesis in Arabidopsis Plant Cell 2005 17 1497 1512 10.1105/tpc.104.030254 15829605 PMC1091770

[B7] MacLean D Jerome CA Brown APC Gary JC Co-regulation of nuclear genes encoding plastid ribosomal proteins by light and plastid signals during seedling development in tobacco and *Arabidopsis* Plant Mol Biol 2008 66 475 490 10.1007/s11103-007-9279-z 18193395

[B8] Pfannschmidt T Plastidial retrograde signalling - a true "plastid factor" or just metabolite signatures? Trends Plant Sci 2010 15 427 435 10.1016/j.tplants.2010.05.009 20580596

[B9] Alberts B Johnson A Lewis J Raf M Roberts K Walter P The molecular biology of the cell 2002 4 Garland Science, Abingdon, New York 1268 pp

[B10] Byrne ME A role for the ribosome in development Trends Plant Sci 2009 14 512 519 10.1016/j.tplants.2009.06.009 19716746

[B11] Schippers JHM Mueller-Roeber B Ribosomal composition and control of leaf development Plant Sci 2010 179 307 315 10.1016/j.plantsci.2010.06.012

[B12] Bailey TL Elkan C Fitting a mixture model by expectation maximization to discover motifs in biopolymers Proc Int Conf Intell Syst Mol Biol 1994 2 28 36 7584402

[B13] Li C Potuschak T Colon-Carmona A Gutierrez RA Doerner P *Arabidopsis *TCP20 links regulation of growth and cell division control pathways Proc Natl Acad Sci USA 2005 102 12978 12983 10.1073/pnas.0504039102 16123132 PMC1200278

[B14] Hughes JD Estep PW Tavazoie S Church GM Computational identification of *cis*-regulatory elements associated with groups of functionally related genes in *Saccharomyces cerevisiae* Journal of Molecular Biology 2000 296 1205 1214 10.1006/jmbi.2000.3519 10698627

[B15] Smith AD Sumazin P Zhang MQ Identifying tissue-selective transcription factor binding sites in vertebrate promoters Proc Natl Acad Sci USA 2005 102 1560 1565 10.1073/pnas.0406123102 15668401 PMC547828

[B16] Huang DW Sherman BT Lempicki RA Systematic and integrative analysis of large gene lists using DAVID Bioinformatics Resources Nat Protoc 2009 4 44 57 19131956 10.1038/nprot.2008.211

[B17] Ma X Zhang K Li X Evolution of *Drosophila *ribosomal protein gene core promoters Gene 2009 432 54 59 10.1016/j.gene.2008.10.025 19059316 PMC3232064

[B18] Chen FW Ioannou YA Ribosomal proteins in cell proliferation and apoptosis Int Rev Immunol 1999 18 429 448 10.3109/08830189909088492 10672495

[B19] Manevski A Bertoni G Bardet C Tremousaygue D Lescure B In synergy with various *cis*-acting elements, plant insterstitial telomere motifs regulate gene expression in *Arabidopsis *root meristems FEBS Lett 2000 483 43 46 10.1016/S0014-5793(00)02056-1 11033353

[B20] Tremousaygue D Garnier L Bardet C Dabos P Herve C Lescure B Internal telomeric repeats and 'TCP domain' protein-binding sites co-operate to regulate gene expression in *Arabidopsis thaliana *cycling cells Plant J 2003 33 957 966 10.1046/j.1365-313X.2003.01682.x 12631321

[B21] Tremousaygue D Manevski A Bardet C Lescure N Lescure B Plant interstitial telomere motifs participate in the control of gene expression in root meristems Plant J 1999 20 553 561 10.1046/j.1365-313X.1999.00627.x 10652127

[B22] Andersson SG Zomorodipour A Andersson JO Sicheritz-Ponten T Alsmark UC Podowski RM Naslund AK Eriksson AS Winkler HH Kurland CG The genome sequence of *Rickettsia prowazekii *and the origin of mitochondria Nature 1998 396 133 140 10.1038/24094 9823893

[B23] Yang D Oyaizu Y Oyaizu H Olsen GJ Woese CR Mitochondrial origins Proc Natl Acad Sci USA 1985 82 4443 4447 10.1073/pnas.82.13.4443 3892535 PMC391117

[B24] Raven JA Allen JF Genomics and chloroplast evolution: what did cyanobacteria do for plants? Genome Biol 2003 4 209 10.1186/gb-2003-4-3-209 12620099 PMC153454

[B25] Cock JM Sterck L Rouze P Scornet D Allen AE Amoutzias G Anthouard V Artiguenave F Aury JM Badger JH Beszteri B Billiau K Bonnet E Bothwell JH Bowler C Boyen C Brownlee C Carrano CJ Charrier B Cho GY Coelho SM Collen J Corre E Silva CD Delage L Delaroque N Dittami SM Doulbeau S Elias M Farnham G Gachon CMM Gschloessl B Heesch S Jabbari K Jubin C Kawai H Kimura K Kloareg B Kupper FC Lang D Bail AL Leblanc C Lerouge P Lohr M Lopez PJ Martens C Maumus F Michel G Miranda-Saavedra D Morales J Moreau H Motomura T Nagasato C Napoli CA Nelson DR Nyvall-Collen P Peters AF Pommier C Potin P Poulain J Quesneville H Read B Rensing SA Ritter A Rousvoal S Samanta M Samson G Schroeder DC Segurens B Strittmatter M Tonon T Tregear JW Valentin K von Dassow P Yamagishi T Van de Peer Y Wincker P The *Ectocarpus *genome and the independent evolution of multicellularity in the brown algae Nature 2010 465 617 621 10.1038/nature09016 20520714

[B26] Dittami SM Scornet D Petit JL Segurens B Silva CD Corre E Dondrup M Glatting KH Konig R Sterck L Rouze P Van de Peer Y Cock JM Boyen C Tonon T Global expression analysis of the brown alga *Ectocarpus siliculosus *(Phaeophyceae) reveals large-scale reprogramming of the transcriptome in response to abiotic stress Genome Biol 2009 10 R66 10.1186/gb-2009-10-6-r66 19531237 PMC2718500

[B27] Merchant SS Prochnik SE Vallon O Harris EH Karpowicz SJ Witman GB Terry A Salamov A Fritz-Laylin LK Marechal-Drouard L Marshall WF Qu LH Nelson DR Sanderfoot AA Spalding MH Kapitonov VV Ren Q Ferris P Lindquist E Shapiro H Lucas SM Grimwood J Schmutz J Chlamydomonas Annotation Team JGI Annotation Team Grigoriev IV Rokhsar DS Grossman AR The *Chlamydomonas *genome reveals the evolution of key animal and plant functions Science 2007 318 245 251 10.1126/science.1143609 17932292 PMC2875087

[B28] Tanay A Regev A Shamir A Conservation and evolvability in regulatory networks: the evolution of ribosomal regulation in yeast Proc Natl Acad Sci USA 2005 102 7203 7208 10.1073/pnas.0502521102 15883364 PMC1091753

[B29] Ihmels J Bergmann S Gerami-Nejad M Yanai I McClellan M Berman J Barkai N Rewiring of the yeast transcriptional network through the evolution of motif usage Science 2005 309 938 940 10.1126/science.1113833 16081737

[B30] Li W Saunders MA The chimpanzee and us Nature 2005 437 50 51 10.1038/437050a 16136126

[B31] Herve C Dabos P Bardet C Jauneau A Auriac MC Ramboer A Lacout F Tremousaygue D In v*ivo *interference with AtTCP20 function induces severe plant growth alterations and deregulates the expression of many genes important for development Plant Physiol 2009 149 1462 1477 10.1104/pp.108.126136 19091878 PMC2649380

[B32] Perry RP The architecture of mammalian ribosomal protein promoters BMC Evol Biol 2005 5 15 10.1186/1471-2148-5-15 15707503 PMC554972

[B33] Richards EJ Ausubel FM Isolation of a higher eukaryotic telomere from *Arabidopsis thaliana* Cell 1988 53 127 136 3349525 10.1016/0092-8674(88)90494-1

[B34] Axelos M Bardet C Liboz T Le Van Thai A Curie C Lescure B The gene family encoding the *Arabidopsis thaliana *translation elongation factor EF-1α: molecular cloning, characterization and expression Mol Gen Genet 1989 219 106 112 2615757 10.1007/BF00261164

[B35] Curie C Liboz T Bardet C Gander E Medale C Axelos M Lescure B *Cis *and *trans*-acting elements involved in the activation of the *Arabidopsis thaliana *A1 gene encoding the translation elongation factor EF-1*α* Nucleic Acids Res 1991 19 1305 1310 10.1093/nar/19.6.1305 1840652 PMC333858

[B36] Liboz T Bardet C Le Van Thai A Axelos M Lescure B The four members of the gene family encoding the *Arabidopsis thaliana *translation elongation factor EF-1α are actively transcribed Plant Mol Biol 1989 14 107 110 10.1007/BF00015660 2101309

[B37] Shewmaker CK Ridge NP Pokalsky AR Rose RE Hiatt WR Nucleotide sequence of an EF-1 alpha genomic clone from tomato Nucleic Acids Res 1990 18 4276 10.1093/nar/18.14.4276 2377481 PMC331215

[B38] Maaloe O Kjeligard NO Control of macromolecular synthesis 1966 WA Benjamin, Inc, New York

[B39] Beemster GTS Fiorani F Inz D Cell cycle: the key to plant growth control? Trends Plant Sci 2003 8 154 158 10.1016/S1360-1385(03)00046-3 12711226

[B40] Tyson JJ Chen K Novak B Network dynamics and cell physiology Nat Rev Mol Cell Biol 2001 2 908 916 10.1038/35103078 11733770

[B41] Bergemann AD Johnson EM The HeLa Pur factor binds single-stranded DNA at a specific element conserved in gene flanking regions and origins of DNA replication Mol Cell Biol 1992 12 1257 1265 1545807 10.1128/mcb.12.3.1257-1265.1992 PMC369558

[B42] Martin W Stoebe B Goremykin V Hansmann S Hasegawa M Kowallik KV Gene transfer to the nucleus and the evolution of chloroplasts Nature 1998 393 162 165 10.1038/30234 11560168

[B43] Woodson JD Chory J Coordination of gene expression between organellar and nuclear genomes Nature 2008 9 383 395 10.1038/nrg2348 PMC4854206 18368053

[B44] Wang Z Zhu XG Chang X Chen YZ Li YX Liu L Though with constraints imposed by endosymbiosis, preferential attachment is still a plausible mechanism responsible for the evolution of the chloroplast metabolic network J Evol Biol 2009 22 71 79 10.1111/j.1420-9101.2008.01621.x 19127608

[B45] Wang Z Zhu XG Chen YZ Li YY Hou J Li Y Liu L Exploring photosynthesis evolution by comparative analysis of metabolic networks between chloroplasts and photosynthetic bacteria BMC Genomics 2006 7 10.1186/1471-2164-7-100 PMC1524952 16646993

[B46] Voss I Koelmann M Wojtera J Holtgrefe S Kitzmann C Backhausen JE Scheibe R Knockout of major leaf ferredoxin reveals new redox-regulatory adaptations in *Arabidopsis thaliana* Physiol Plant 2008 133 584 598 10.1111/j.1399-3054.2008.01112.x 18494733

[B47] Arai T Kelly VP Minowa O Noda T Nishimura S High accumulation of oxidative DNA damage, 8-hydroxyguanine, in Mmh/Ogg1 deficient mice by chronic oxidative stress Carcinogenesis 2002 23 2005 2010 10.1093/carcin/23.12.2005 12507922

[B48] Ledford HK Niyogi KK Singlet oxygen and photo-oxidative stress management in plants and algae Plant Cell and Environ 2005 28 1037 1045 10.1111/j.1365-3040.2005.01374.x

[B49] Niyogi KK Photoprotection revisited: Genetic and molecular approaches Annu Rev Plant Physiol Plant Mol Biol 1999 50 333 359 10.1146/annurev.arplant.50.1.333 15012213

[B50] Pregueiro AM Liu QY Baker CL Dunlap JC Loros JJ The *Neurospora *checkpoint kinase 2: A regulatory link between the circadian and cell cycles Science 2006 313 644 649 10.1126/science.1121716 16809488

[B51] Unsal-Kacmaz K Mullen TE Kaufmann WK Sancar A Coupling of human circadian and cell cycles by the timeless protein Mol and Cell Biol 2005 25 3109 3116 10.1128/MCB.25.8.3109-3116.2005 PMC1069621 15798197

[B52] Medina M Genomes, phylogeny, and evolutionary systems biology Proc Natl Acad Sci USA 2005 102 6630 6635 10.1073/pnas.0501984102 15851668 PMC1131869

[B53] Hedges SB The origin and evolution of model organisms Nat Rev Genet 2002 3 838 849 12415314 10.1038/nrg929

[B54] Nielsen H Engelbrecht J Brunak S von Heijne G Identification of prokaryotic and eukaryotic signal peptides and prediction of their cleavage sites Protein Eng 1997 10 1 6 10.1093/protein/10.1.1 9051728

[B55] Nakao A Yoshihama M Kenmochi N RPG: the ribosomal protein gene database Nucleic Acids Res 2004 32 D168 D170 10.1093/nar/gkh004 14681386 PMC308739

[B56] Yamaguchi K Beligni MV Prieto S Haynes PA McDonald WH Yates JR III Mayfield SP Proteomic characterization of the *Chlamydomonas reinhardtii *chloroplast ribosome: identification of proteins unique to the 70S ribosome J Biol Chem 2003 278 33774 33785 10.1074/jbc.M301934200 12826678

[B57] Yamaguchi K Prieto S Beligni MV Haynes PA McDonald WH Yates JR III Mayfield SP Proteomic characterization of the small subunit of *Chlamydomonas reinhardtii *chloroplast ribosome: identification of a novel S1 domain-containing protein and unusually large orthologs of bacterial S2, S3, and S5 The Plant Cell 2002 14 2957 2974 10.1105/tpc.004341 12417713 PMC152739

[B58] Schmid M Davison TS Henz SR Pape UJ Demar M Vingron M Scholkopf B Weigel D Lohmann JU A gene expression map of *Arabidopsis thaliana *development Nat Genet 2005 37 501 506 10.1038/ng1543 15806101

[B59] Baerenfaller K Grossmann J Grobei MA Hull R Hirsch-Hoffmann M Yalovsky S Zimmermann P Grossniklaus U Gruissem W Baginsky S Genome-scale proteomics reveals *Arabidopsis thaliana *gene models and proteome dynamics Science 2008 320 938 941 10.1126/science.1157956 18436743

[B60] Hyams J Davies DR The induction and characterization of cell wall mutants of *Chlamydomonas reinhardi* Mutat Res 1972 14 381 389

[B61] Gorman DS Levine RP Cytochrome f and plastocyanin: their sequence in the photosynthetic electron transport chain of *Chlamydomonas reinhardi* Proc Natl Acad Sci USA 1965 54 1665 1669 10.1073/pnas.54.6.1665 4379719 PMC300531

[B62] Vila M Couso I Leon R Carotenoid content in mutants of the chlorophyte *Chlamydomonas reinhardtii *with low expression levels of phytoene desaturase Proc Biochem 2008 43 1147 1152 10.1016/j.procbio.2008.06.014

[B63] Gonzalez-Ballester D Camargo A Fernandez E Ammonium transported genes in *Chlamydomonas*: the nitrate-specific regulatory gene *Nit2 *is involved in *Amt1*;1 expression Plant Mol Biol 2004 56 863 878 10.1007/s11103-004-5292-7 15821986

