# Supplementary material for: Synchronization of cytoplasmic and transferred mitochondrial ribosomal protein gene expression in land plants is linked to Telo-box motif enrichment
Source: BMC Evol Biol. 2011 Jun 13;11:161. doi: 10.1186/1471-2148-11-161 (PMC3212954; doi:10.1186/1471-2148-11-161)
Supplement: Additional file 1 — Table S1 and Figures S1-S12. Table S1: Catalogs of cytoplasmic and transferred organelle ribosomal protein genes. Figure S1: Occurrence frequency of RPGs in 132 plant chloroplast and 25 plant mitochondrial genomes. (A) More than half of chloroplast RPGs are absent in most chloroplast genomes of 132 plants surveyed (green dash line), providing a raw estimate of the lower bound of transfer frequency of cpRPGs. (B) Similarly, there are over 50 RPGs in Rickettsia prowazekii str. Madrid E (α-proteobacteria, an ancient cousin of mitochondrion), whereas only 18 mtRPGs can be found in the union of all 25 plant mitochondrial genomes and most mtRPGs are absent in over half studied species (red dash line). Genes with zero occurrence frequency are present in corresponding ancient cousin but absent in chloroplast/mitochondria of all studied plants. Figure S2: Protein expression correlation of mtRPGs, cpRPGs and euRPGs in A. thaliana. Each element of the matrix represents the Pearson's correlation coefficient between the expression profiles of two ribosomal proteins. Figure S3: RNA expression correlation of mtRPGs, cpRPGs and euRPGs in different tissues of several developmental stages in A. thaliana. 7th day (A), 17th day (B), 21st day (C) and 8th week (D) corresponds to stage of seedling, leaf, flower and seed of A. thaliana, respectively. Each element of the matrix represents the Pearson's correlation coefficient of the expression profiles of each two RPGs. Color code is illustrated in bottom panel. Figure S4: Putative promoter motifs only enriched in one of cpRPGs, mtRPGs, and euRPGs in A. thaliana. The number on the left of each logo is E-value of MEME prediction. Figure S5: Putative promoter motifs predicted by AlignACE and DME. The number on the left of each logo is score of AlignACE and DME prediction. Figure S6: Expression correlation between RPGs with telo-box and those without telo-box. Telo-box here indicates the sequence AAACCCT or AACCCTA. The error-bar is the standa [file 1471-2148-11-161-S1.DOC]

**Additional File 1: Table S1 and Figure S1-S12**

**Table S1: Catalogs of cytoplasmic and transferred organelle ribosomal protein genes**

| Species | Compartment^*^ | Num. of RPGs | Species | Compartment^*^ | Num. of RPGs |
| --- | --- | --- | --- | --- | --- |
| *A. thaliana* | C | 42 | *S. moellendorffii* | C | 20 |
|  | M | 47 |  | M | 29 |
|  | E | 125 |  | E | 91 |
| *P. trichocarpa* | C | 44 | *P. patens* | C | 27 |
|  | M | 33 |  | M | 23 |
|  | E | 206 |  | E | 146 |
| *M. truncatula* | C | 19 | *C. reinhardtii* | C | 33 |
|  | M | 23 |  | M | 16 |
|  | E | 71 |  | E | 54 |
| *O. sativa* | C | 36 | *E. siliculosus* | C | 15 |
|  | M | 36 |  | M | 6 |
|  | E | 77 |  | E | 76 |

^*^ The symbols C, M and E in column of compartment indicate chloroplast, mitochondrion and cytoplasm, respectively.


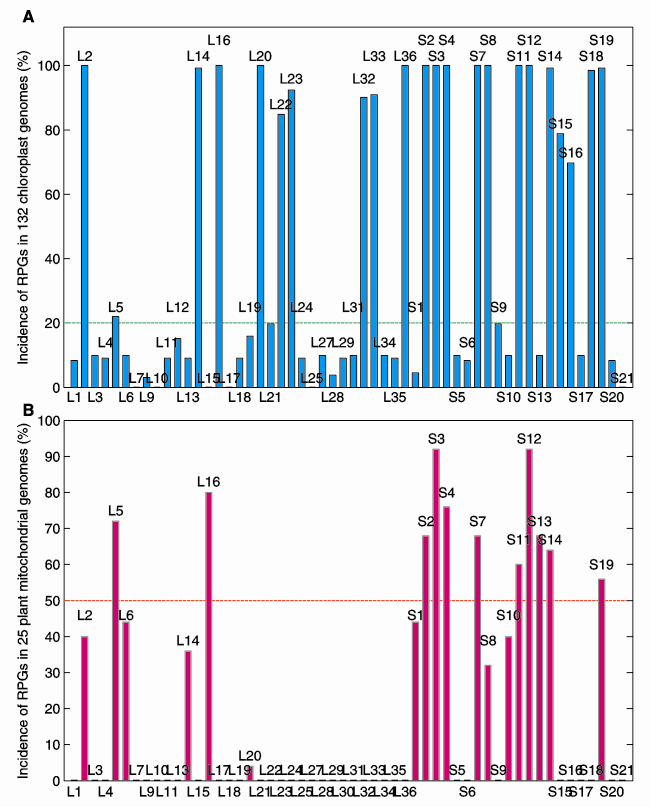


**Figure S1**


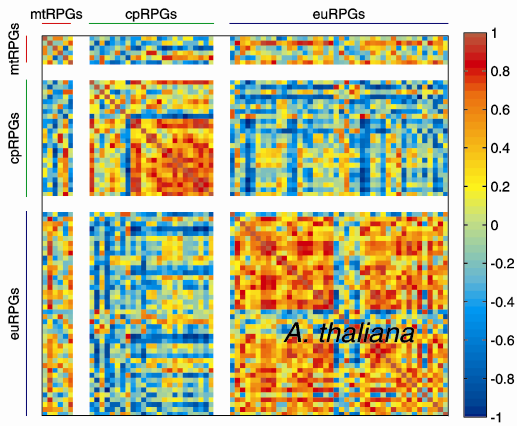


**Figure S2**


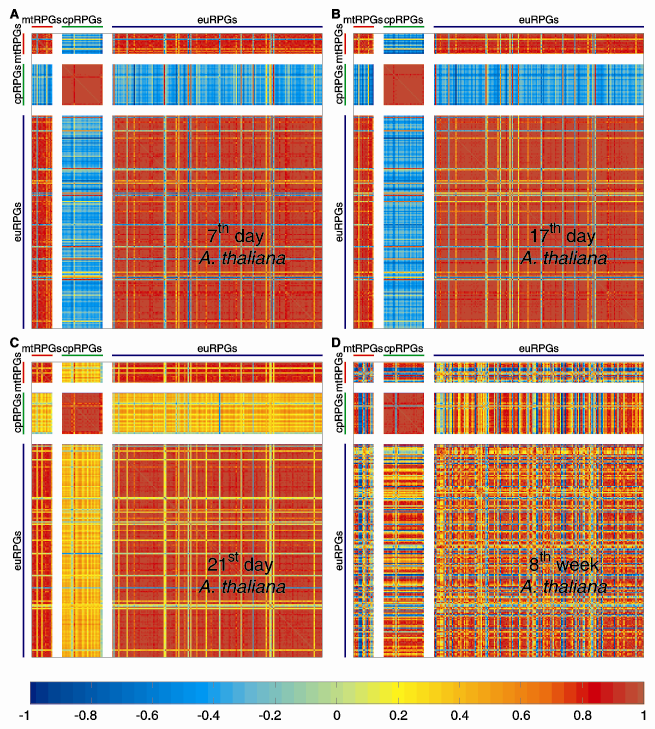


**Figure S3**


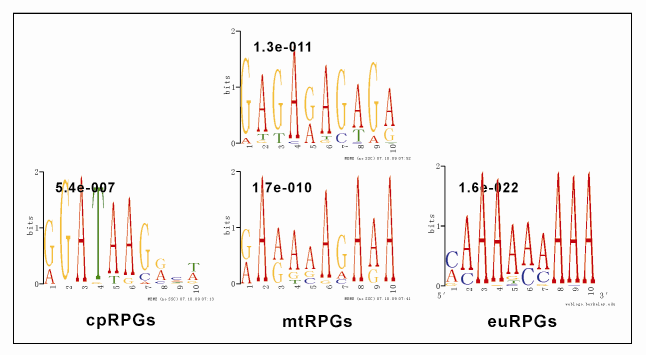


**Figure S4**


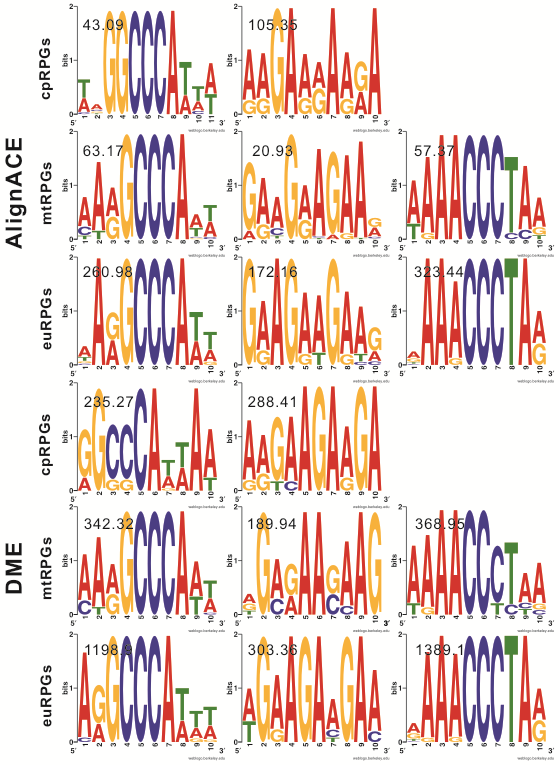


**Figure S5**


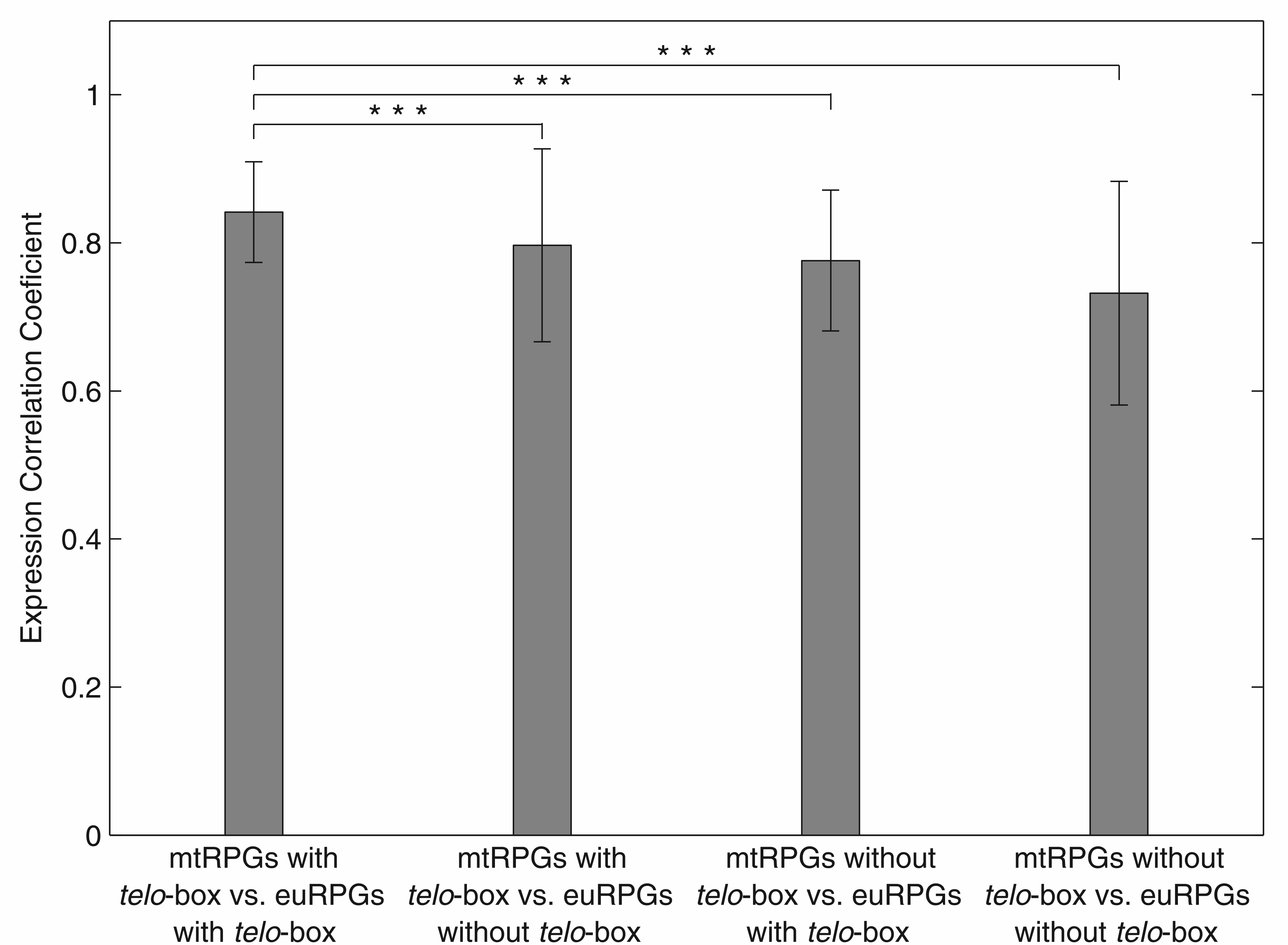


**Figure S6**


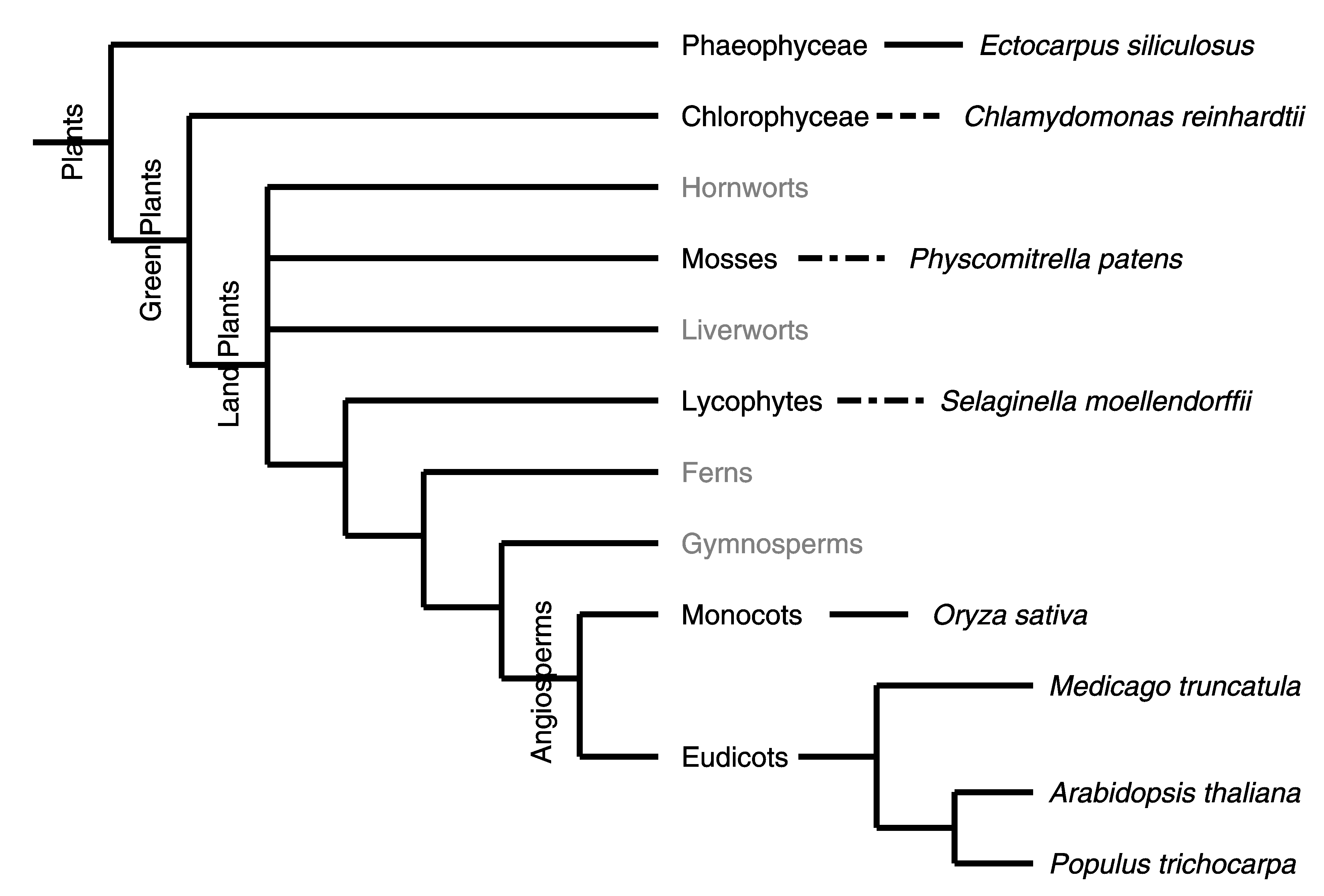


**Figure S7**


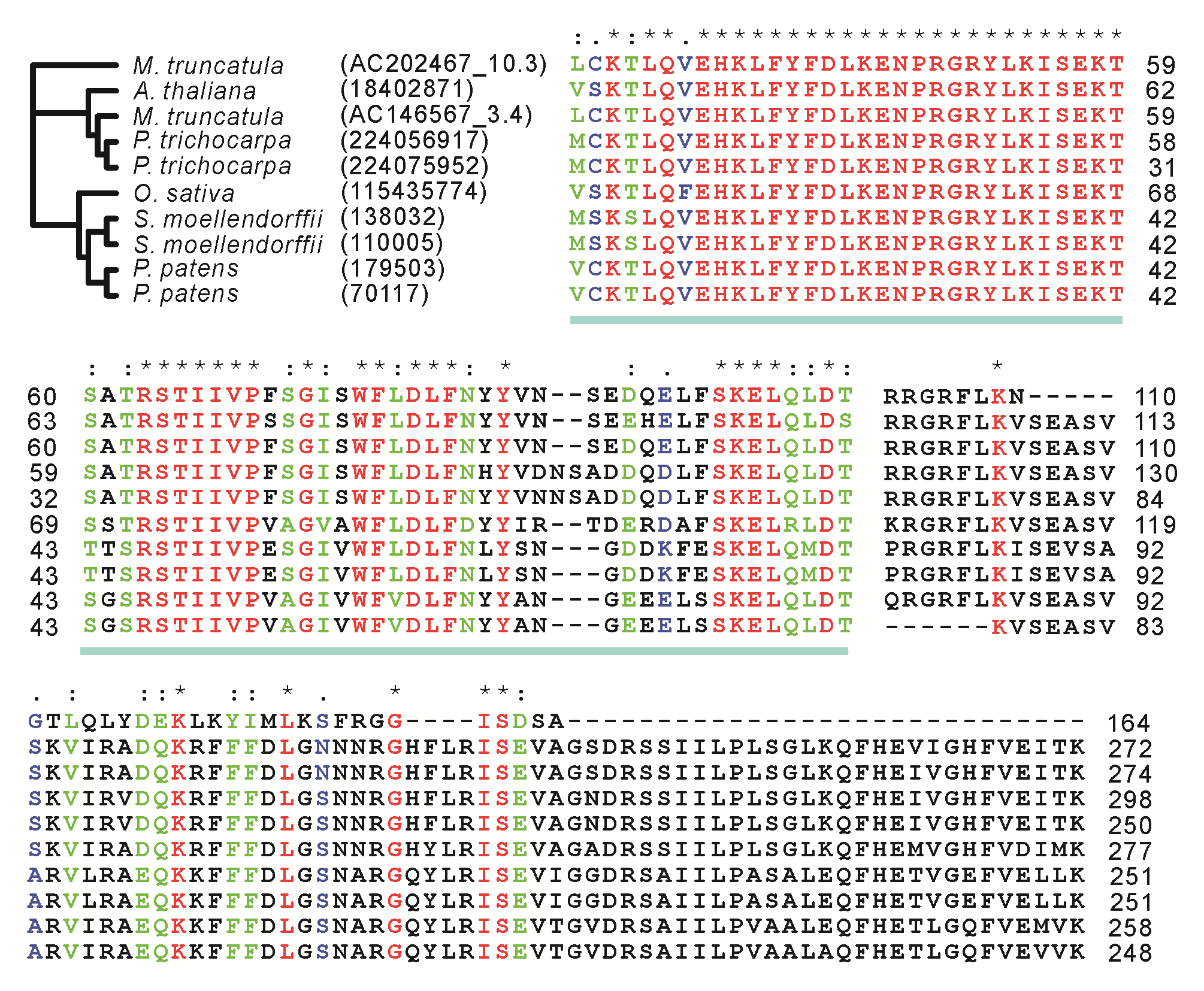


**Figure S8**


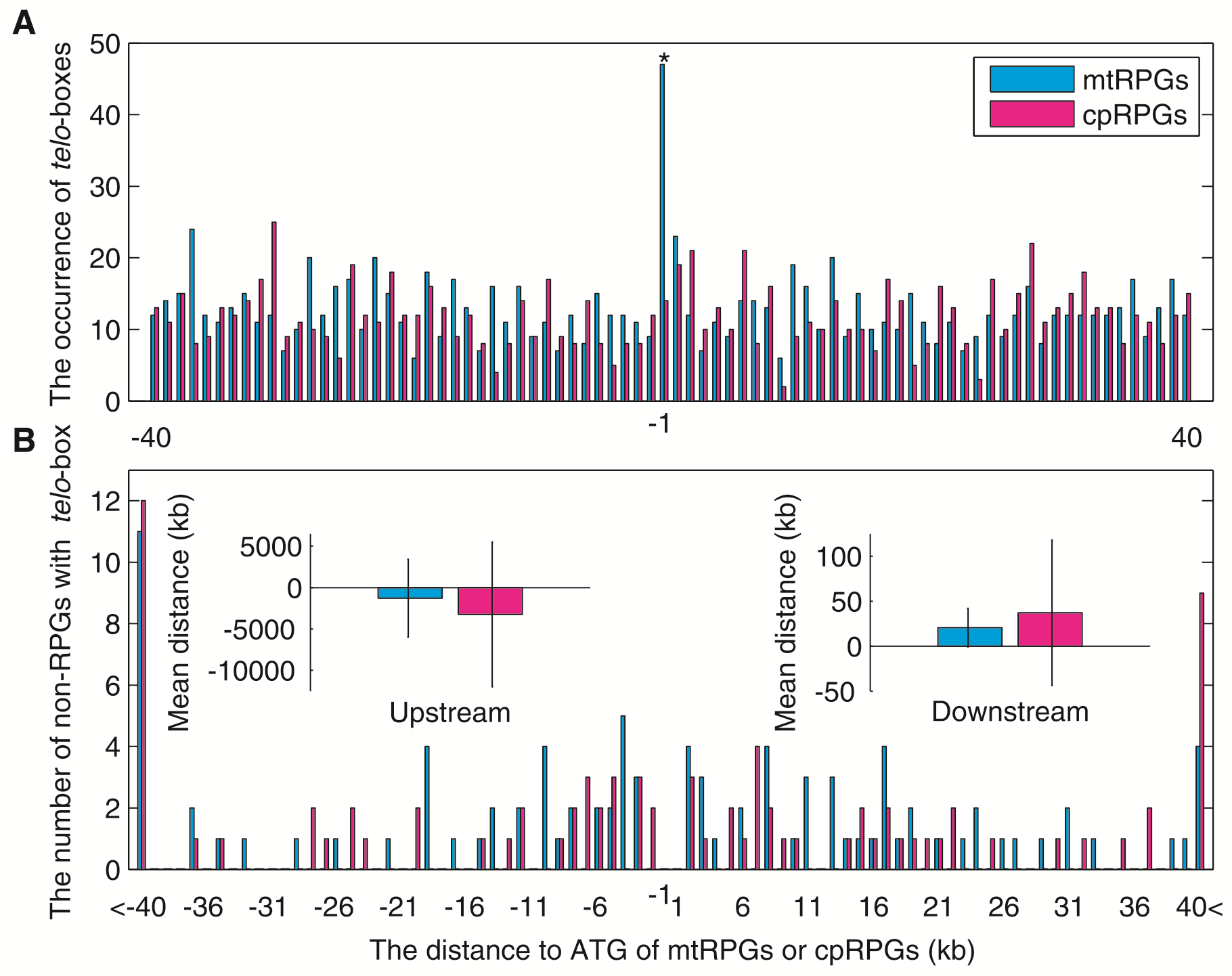


**Figure S9**


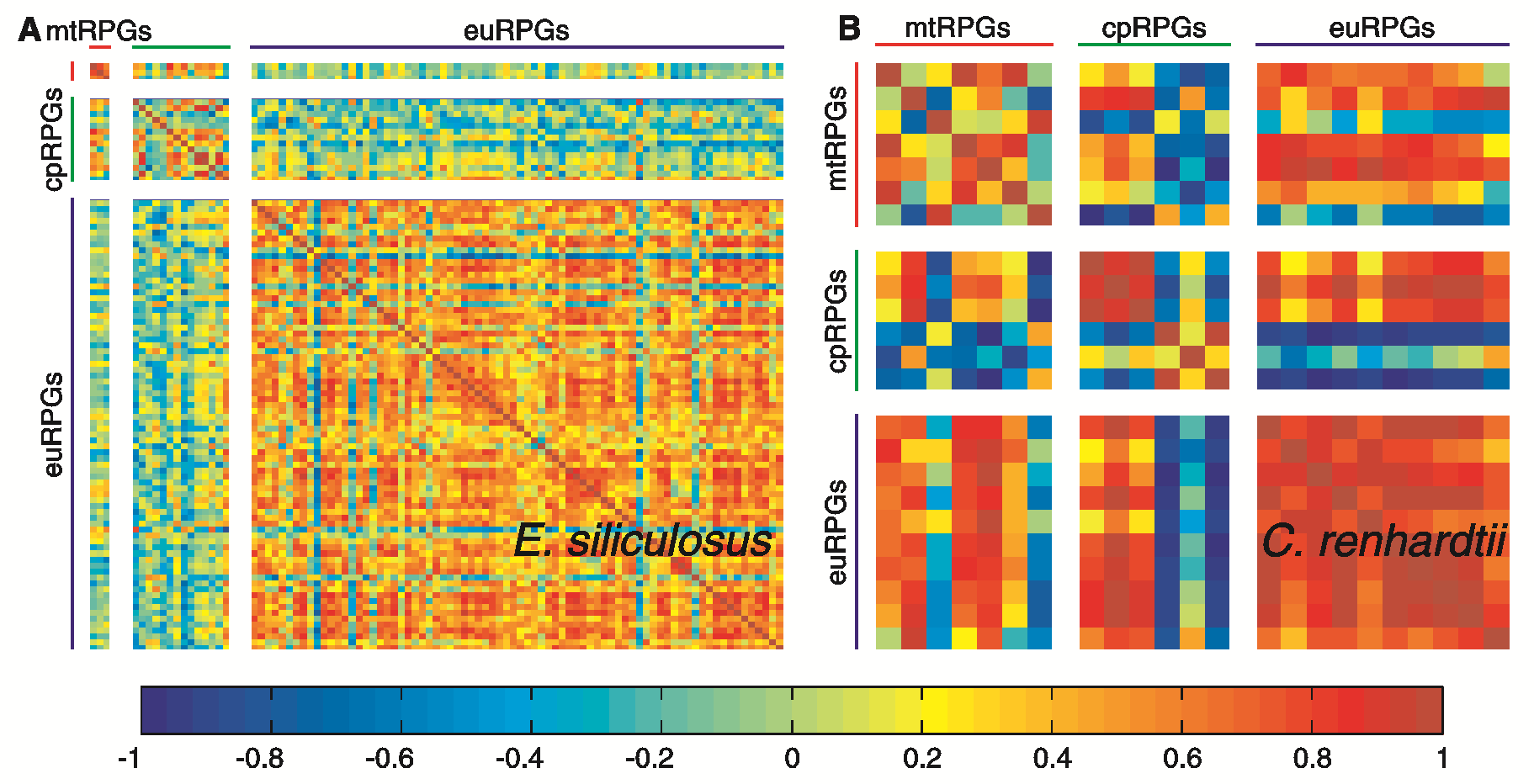


**Figure S10**


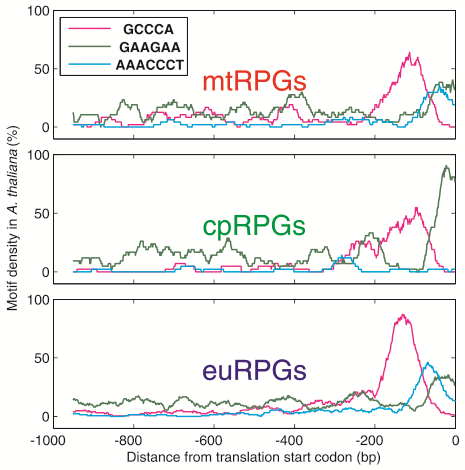


**Figure S11**


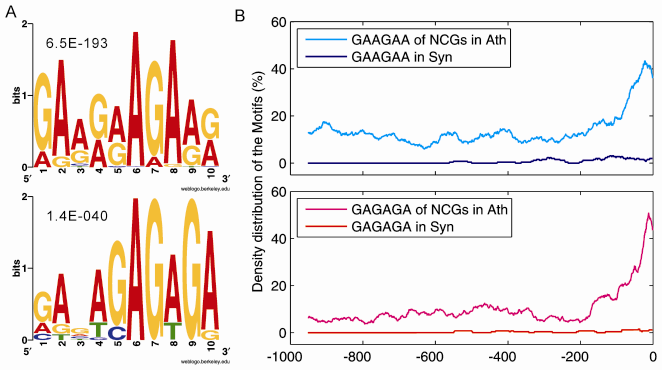


**Figure S12**
